# Negative Regulation of p21^Waf1/Cip1^ by Human INO80 Chromatin Remodeling Complex Is Implicated in Cell Cycle Phase G2/M Arrest and Abnormal Chromosome Stability

**DOI:** 10.1371/journal.pone.0137411

**Published:** 2015-09-04

**Authors:** Lingling Cao, Jian Ding, Liguo Dong, Jiayao Zhao, Jiaming Su, Lingyao Wang, Yi Sui, Tong Zhao, Fei Wang, Jingji Jin, Yong Cai

**Affiliations:** 1 School of Life Sciences, Jilin University, Changchun, Jilin, China; 2 National Engineering Laboratory for AIDS Vaccine, Jilin University, Changchun City, Jilin, China; 3 Key Laboratory for Molecular Enzymology and Engineering, the Ministry of Education, Jilin University, Changchun City, Jilin, China; German Cancer Research Center, GERMANY

## Abstract

We previously identified an ATP-dependent human Ino80 (INO80) chromatin remodeling complex which shares a set of core subunits with yeast Ino80 complex. Although research evidence has suggested that INO80 complex functions in gene transcription and genome stability, the precise mechanism remains unclear. Herein, based on gene expression profiles from the INO80 complex-knockdown in HeLa cells, we first demonstrate that INO80 complex negatively regulates the p21^Waf1/Cip1^ (p21) expression in a p53-mediated mechanism. In chromatin immunoprecipitation (ChIP) and a sequential ChIP (Re-ChIP) assays, we determined that the INO80 complex and p53 can bind to the same promoter region of p21 gene (-2.2kb and -1.0kb upstream of the *p21* promoter region), and p53 is required for the recruitment of the INO80 complex to the p21 promoter. RNAi knockdown strategies of INO80 not only led to prolonged progression of cell cycle phase G2/M to G1, but it also resulted in abnormal chromosome stability. Interestingly, high expression of p21 was observed in most morphologically-changed cells, suggesting that negative regulation of p21 by INO80 complex might be implicated in maintaining the cell cycle process and chromosome stability. Together, our findings will provide a theoretical basis to further elucidate the cellular mechanisms of the INO80 complex.

## Introduction

Ino80 chromatin remodeling complex, a member of Ino80 subfamily of the SWI/SNF chromatin remodeling superfamily, is highly conserved from *Saccharomyces cerevisiae* to human [1]. We previously identified a human ATP-dependent chromatin remodeling complex, which shares eight core subunits with yeast Ino80 complex, including a SNF2 ATPase-INO80 catalytic subunit, actin-related proteins Arp4, Arp5 and Arp8, Tip49a and Tip49b AAA^+^ ATPases, and hIes2 and hIes6 [2,3]. Recently, increasing evidence has suggested that the INO80 complex is involved in many biological processes in cells including gene transcription, DNA damage repair, telomere maintenance, and embryonic stem cells (ESCs) self-renewal [3–7].

Like the other chromatin remodelers, INO80 complex possesses ATPase and DNA nucleosomal sliding activities. Using the energy of ATP hydrolysis, INO80 can alter histone-DNA interactions, causing nucleosomes to move in *cis* (sliding) [8]. Thus, INO80 plays a role in concert to organize chromatin structure by depositing, moving, evicting, or selectively altering nucleosomes in an ATP-dependent manner [9]. Research into the functions of INO80 in replication, DNA damage repair and gene transcription in higher eukaryotes has been rather incomplete, but increasing data reveals that INO80 complex plays an important role in those processes. Depletion of Ino80 not only resulted in slower growth and reduced S-phase progression, but also led to defective telomere replication, impaired DNA repair and hydroxyurea (HU) sensitivity [4,5,10]. Recent electron microscopy (EM) studies showed that the Ino80 complex formed an elongated embryo-like shape with head-neck-body-foot topology in yeast [11,12]. Although the complete crystal structure of INO80 complex is still unknown, three modules that assemble on three distinct domains of the INO80 protein have been reported [13]. All shared core subunits composed of two modules and assembled on the conserved helicase-SANT-associated/post-HSA (HSA/PTH) and ATPase domains of INO80 protein. Both HSA/PTH and ATPase domains are essential for catalyzing the ATP-dependent nucleosome remodeling activity of the INO80 complex. Lacking Arp4 and Arp8 in yeast, Ino80 complex is defective in DNA binding, ATPase, and nucleosome remodeling activities [14]. In human cells, INO80 complex is recruited to DNA damage sites in an Arp8-dependent manner [15].

Chromatin remodelers play key roles in maintaining normal dynamic changes of chromatin structure in cells [16]. However, dynamic changes of chromatin formation are often affected by many factors during the cell cycle, of which DNA damage and its repair processes are the most important factors. Evidence from yeast and human cell research suggests that the Ino80 complex participates in DNA damage repair [4,6], and the recruitment of Ino80 complex to DNA double strand breaks is in a cell cycle-dependent manner [17]. Further studies revealed that checkpoint kinases and the Ino80 complex enhance global chromatin mobility in response to DNA damage [18], suggesting the roles of Ino80 complex in DNA damage repair and in cell cycle checkpoint control.

CDKN1A (p21^Waf1/Cip1^) is a universal inhibitor of cyclin kinases which controls cell cycle by activating and/or inactivating the cyclin-dependent kinases (CDKs) [19,20]. It is well known that p21 is both a classical tumor suppressor and an oncogene, and is involved in many cellular processes [21]. Accumulation of cytoplasmic p21 has been reported in many cancers and is associated with tumor aggressiveness, metastasis, and prognosis [22]. Activated tumor suppressor p53 can bind directly to two highly conserved p53 response elements (PRE) in the p21 promoter and induce transcriptional activation of the *p21* gene [23]. Over the past few decades, research in the mechanisms regulating p21 transcription has been investigated mostly in cells that have defective cellular senescence pathways [24]. Interestingly, recent findings indicate that epigenetic regulators might be involved in transcriptional regulation of p21. For instance, SWI2/SNF2-related chromatin remodeling complexes including p400 and SRCAP are localized to the p21 promoter region near the p53 binding sites, and negatively regulate p21 transcription in a p53-dependent manner [25,26]. Moreover, p400 chromatin remodeler plays a key role in the deposition of the histone variant H2AZ within the p21 promoter, therefore repressing p21 gene expression [27].

Although it has been known that INO80 complex functions in DNA damage repair and therefore leads to delayed or arrested cell cycle, the precise mechanisms, especially its target genes, remains unclear. In an effort to resolve which genes are responsible for INO80 involved cellular functions including cell cycle regulation, we carried out the gene expression profiles in INO80 core subunits knockdown in HeLa cells. With combined methods of ChIP and Re-ChIP assays, RNAi knockdown, flow cytometry, immunofluorescence staining, RT-PCR and Western blots, we first demonstrate: 1) the p21, an inhibitor of cyclin kinases, is negatively regulated by the INO80 complex in a p53-mediated mechanism; 2) INO80 complex and p53 co-occupy the p21 promoter (-2.2 kb and -1.0 kb upstream of the p21 promoter region), the existence of p53 is required for recruiting the INO80 complex to the p21 promoter; and 3) INO80 complex might be implicated in maintaining the normal cell cycle and genome stability. Our results shed light on how INO80 complex is involved in cell cycle and in the maintenance of chromosome stability.

## Materials and Methods

### Antibodies

Anti-YY1 (H414, sc-1703X) rabbit polyclonal antibody, anti-Myc (9E10) and anti-α-tubulin (sc-58666) monoclonal antibodies, rabbit total IgG (sc-2027) and mouse total IgG (sc-2025) were obtained from Santa Cruz Biotechnology (U.S.A.). Anti-Flag (M2)-agarose and anti-Flag M2 (F3165) monoclonal antibody were from Sigma (U.S.A.). Anti-p21 (10355-1-AP) was purchased from Proteintech^TM^ Group (China, Wuhan). Anti-p53-15P and anti-p53-33P rabbit polyclonal antibodies were gained from Dingguo Changsheng Biotechnology Co. LTD. (China, Beijing). Anti-INO80 (residue 1–526 aa), anti-hArp8, anti-hIes6, anti-hIes2, anti-p53, and anti-GAPDH rabbit polyclonal antibodies were raised against bacterially expressed proteins (Jilin University). Anti-p53 monoclonal antibody was from ZYMED (13–4000). Anti-pericentrin (ab4448) rabbit polyclonal antibody was from Abcam (UK).

### Cell culture/Maintainence

Human embryonic kidney (HEK) 293T cells and HeLa cells were maintained in Dulbecco’s modified Eagle’s Medium (DMEM) (Gibco, Life Technologies^TM^, U.S.A.) supplemented with 10% fetal bovine serum (KangYuan Biology, China) and 1% Penicillin-Streptomycin mixture (Thermo Fisher Scientific, U.S.A) at 37°C in the presence of 5% CO_2_. The human HCT116 colon carcinoma cells including p53 wild type (p53+/+) and p53-null (p53-/-) (kindly gifted by Dr. Yinghua Jin (Jilin University) were cultured in Iscove’s Modifies Dulbecco’s Medium (IMDM) (Gibco, Life Technologies^TM^, U.S.A.) containing 10% fetal bovine serum (Hyclone, Thermo Fisher Scientific, U.S.A).

### Chemical treatment

Camptothecin (CPT) (Cat. C991, Sigma) was dissolved in dimethyl sulfoxide (DMSO) and prepared into 5 mg/ml concentration for storage. The final concentration of CPT in cell culture medium is 5μM. 1M/L hydroxyurea (HU) (Cat. H8267, Sigma) was prepared with double distilled water (ddH_2_O) as a stock solution. Doxorubicin hydrochloride (D1515) from Sigma was dissolved in DMSO. The final concentration of doxorubicin in cell culture medium is 0.5μM.

### Constructions of plasmids and transient transfection

Flag-tagged full length (FL) of p53 (GI:371502114) were constructed by inserting cDNAs into the XhoI/BamHI sites of pcDNA3.1(-). The pBS/U6 vector encoding INO80 shRNA (pBluescript-shINO80) was a kindly gift from Dr. Yang Shi in Harvard Medical School [25,17]. For transfection of either Flag-tagged p53FL proteins or INO80-shRNA plasmids, cells were seeded in 6-well tissue culture plates (~3 × 10^5^ cells/well). Next day, 2μg plasmids were then transiently transfected using 6μL polyethylenimine (PEI) (Cat. 23966, Polysciences) according to the manufacturer’s recommendation. After 48 hours post transfection, whole cell lysates were prepared and subjected to 12% SDS-PAGE gel electrophoresis. The proteins were analyzed by Western blot with appropriate antibodies.

### Reverse transcription PCR (RT-PCR)

Total RNA was isolated using RNAiso Plus (Takara, Cat.No-D9109). Total RNA (1μg) from each sample was used as a template to produce cDNA with PrimeScript 1st Strand cDNA Synthesis Kit (TAKARA). *INO80*, *Arp8*, *Ies6*, *Ies2*, *CCNG1*, *CCNC*, *CCNH*, *CDK6*, *CDC34*, *p15*, *ATMIN*, *BCCIP*, *CDK2*, *CycE1*, *MAD2L*, *p21*, *p53*, and *GAPDH* mRNA was measured by quantitative real time PCR (qPCR) with Eco^TM^ Real-Time PCR System (Illumina, Gene Company Limited). All PCR reactions were performed under the following protocol: initial denaturation step was at 95°C for 30 seconds, followed by 40 cycles of denaturation at 95°C for 5 seconds, annealing at 60°C for 30 seconds. The specific mRNA was measured by qPCR with indicated RT-primers (**Table 1**).

**Table 1:** RT-qPCR primer sets used in paper.

Primers	Directions	Sequences	Size (bp)
hIno80	forward	5'-CGGAATCGGCTTTTGCTA-3'	292
	reverse	5'-TGTCGGCTGGTCAGTTGG-3	
hArp8	forward	5'-CCAGGCTGAGAAGGGTGATA-3'	200
	reverse	5'-GCAGGAAGAGTGTCTGTGGC-3'	
hIes6	forward	5'-ATGGCGGCGCAAATTCCAAT-3'	247
	reverse	5'-AATGGCAAAGGTTTGGCAGC-3'	
hIes2	forward	5'-GGAGAAGCCCTGGAGTTGAG-3'	199
	reverse	5'-GGAACACTCTTGGTCCCCAG-3'	
GAPDH	forward	5'-ATCACTGCCACCCAGAAGAC-3'	443
	reverse	5'-ATGAGGTCCACCACCCTGTT-3'	
p21	forward	5'-ATGTGGACCTGTCACTGTCTTG-3'	140
	reverse	5'-CGTTTGGAGTGGTAGAAATCTG-3'	
BCCIP	forward	5'-TCAAGAGTTGGTTCTACGCTTC-3'	111
	reverse	5'-CATGGGCAGAGCGATCTGT-3'	
ATMIN	forward	5'-CAACCAATCCCTAGACCAGACA-3'	181
	reverse	5'-GCATCACGGGTAGTTTAACCAAA-3'	
CDK6	forward	5'-CCAGATGGCTCTAACCTCAGT-3'	152
	reverse	5'-AACTTCCACGAAAAAGAGGCTT-3'	
CCNG1	forward	5'-GAGTCTGCACACGATAATGGC-3'	168
	reverse	5'-GTGCTTGGGCTGTACCTTCA-3'	
CCNC	forward	5'-CCTTGCATGGAGGATAGTGAATG-3'	62
	reverse	5'-AAGGAGGATACAGTAGGCAAAGA-3'	
CCNH	forward	5'-AGGCACTTGAACAGATACTGGA-3'	132
	reverse	5'-CCAATATGGGATAGCGGGTCT-3'	
CDC34	forward	5'-GACGAGGGCGATCTATACAACT-3'	113
	reverse	5'-GAGTATGGGTAGTCGATGGGG-3'	
p15	forward	5'-GGGAGGGTAATGAAGCTGAG-3'	97
	reverse	5'-GGCCGTAAACTTAACGACACT-3'	
MAD2L1	forward	5'-TTCTCATTCGGCATCAAC-3'	224
	reverse	5'-TCCAGGACCTCACCACTT-3'	
CDK2	forward	5’-CCAGGAGTTACTTCTATGCCTGA-3'	90
	reverse	5’-TTCATCCAGGGGAGGTACAAC-3'	
p53	forward	5’-CAGCACATGACGGAGGTTGT-3'	125
	reverse	5’-TCATCCAAATACTCCACACGC-3'	
P53-C	forward	5’-ACTAAGCGAGCACTGCCCA-3'	300
	reverse	5’-TCAGTCTGAGTCAGGCCCTT-3'	
P53-N	forward	5’-ATGGAGGAGCCGCAGTCAG-3'	264
	reverse	5’-TGCTCCCTGGGGGCAGCTCG-3'	
CyclinE1	forward	5’-ACTCAACGTGCAAGCCTCG-3'	141
	reverse	5’-GCTCAAGAAAGTGCTGATCCC-3'	
Bax	forward	5’-CTCAGGATGCGTCCACCAAGAA-3'	287
	reverse	5’-CTCCCGGAGGAAGTCCAATGTC-3'	

### DNA microarray and statistical analysis

HeLa cells were cultured in 6-well tissue culture plates (~2 × 10^5^ cells/well) in DMEM medium containing 10% fetal bovine serum. Arp8 siRNA (sc-60072) was obtained from Santa Cruz, while all specific siRNAs including non-targeting siRNA (D-001206), INO80 siRNA (D-004176), Ies6 siRNA (D-019327) and Ies2 siRNA (D-009848) SMART pool were from Dharmacon (U.S.A.). The cells were transiently transfected with 10~20pmol specific siRNAs using Lipofectamine RNAiMAX transfection kit (Invitrogen, Cat.No-864425) following the manufacturer’s instruction. 48 hours after siRNA transfection, cells were then stored in an RNA hold solution (ER501–01, Beijing Transgen Biotech Co., Ltd.) and sent to EMTD Science and Technology Development Co., Ltd. (Beijing, China) for DNA microarray analysis. The Illumina microarray datasets were accessible from the National Center for Biotechnology Information Gene Expression Omnibus (GEO) data repository (http://www.ncbi.nlm.nih.gov/geo/) using the series accession number GSE68655. In order to obtain functional annotation of INO80-regulated genes in our study, the online biological classification tool DAVID (Database for Annotation, Visualization and Integrated Discovery) was used to perform enrichment analysis [28,29]. Furthermore, DAVID was utilized to conduct Gene Ontology (GO) [30] function and Kyoto Encyclopedia of Genes and Genomes (KEGG) [31] pathway enrichment analysis of differentially expressed genes (DEGs). During the analysis, *p* < 0.05 and FDR < 0.02 genes used in the annotation were defined as statistically significant.

### Flow cytometry analysis

HEK293T cells were grown to 50–60% confluence in DMEM medium containing 10% FBS. In order to determine the effect of INO80 on cell cycle, cells were transfected with pBluescript-vector (pBS-Vector) or pBlueScript-shINO80 (pBS-shINO80) plasmids. Cells were harvested 48 hours after transfection for flow cytometry analysis. In the cell cycle arrest experiment, cells were synchronized by treatment with 1mM hydroxyurea (HU). Cells were harvested by trypsinization 0, 2, 4, 6h, 8h and 12 hours after removal of HU. 10^6^ cells with or without synchronization were suspended as single cell dispersions in 70% ethanol at -20°C at least 4 hours. After ethanol fixation, cells were centrifuged at 300 × g for 5 minutes and ethanol removed. Cells were washed twice with PBS buffer and then re-suspended in 300μl PBS containing 0.1% (v/v) Triton X-100 (Sigma, Cat. T8787), 0.3 mg/ml DNase-free, RNase A (Sigma, Cat. R5500), 50μg/ml propidium iodide (Beijing Dingguo Cat. CF0031), then were incubated at 37°C for 1 hour. Data collection was performed using EPICSXL^TM^ Cytometers (Beckman Coulter). Acquired data was analyzed using ModFit LT software (Verity Software House, U.S.A.).

### Immunofluorescence Staining

Cells were cultured and grown to ~30% confluence in 24-well plates containing a coverslip (8D1007, Nest) on each well. Cells were then transfected with INO80 siRNA or INO80 shRNA and hArp8 siRNA using RNAiMAX (Invitrogen). 48 hours later, cells were washed by PBS buffer, fixed with 4% Paraformaldehyde (PFA) for 15 minutes at room temperature, and permeabilized with 0.5% TritonX-100 in PBS buffer for 5 minutes, followed by blocking with 1% bovine serum albumin in PBS for an hour at 37°C. Then, cells were washed for 5 minutes in PBS-T three times, and incubated with p21, α-tubulin or pericentrin primary antibodies (1:200–500) at room temperature, then stained with FITC-conjugated secondary antibodies (rabbit/green: 1:300, sc-2012; mouse/green: 1:300, sc-2010; rabbit/red: 1:500, A10040). Cell nuclei were stained by Vectashield with DAPI (Vecter Laboraries,Inc. Cat.No-H-1200). Fluorescence images were observed with Olympus BX40F Microscope (Olympus Corporation).

### Chromatin Immunoprecipitation (ChIP) Assay

One 10 cm dish (1 × 10^7^) of HEK293T, HeLa, HCT116 p53 wild type (p53+/+) or HCT116 p53-null (p53-/-) cells grown to 80% of confluence were used for each ChIP. ChIP and Re-ChIP assays were carried out essentially as described using INO80, YY1 and p53 antibodies [3,32]. ChIP’d DNA was subjected to qPCR. Each experiment was performed 2–3 independent times. Both of ChIP and no-Ab signal were normalized to total input. Six primer sets were used in quantitative real time PCR (qPCR). The primer sets for qPCR on the promoter region of p21 were as follows: p21 -2.5kb (-2494bp~-2380bp), 5’-ACATTGTTCCCAGCACTTCC-3’ (forward) and 5’-TAGGGGAATGGTGAAAGGTG-3’ (reverse); -2.2kb (-2261bp~-2132bp), 5’- CTGTGGCTCTGATTGGCTTT-3’ (forward) and 5’-CTCCTACCATCCCCTTCCTC-3’ (reverse); -1.6kb (-1696bp~-1554bp), 5’-TCTGGGGTTTAGCCACAATC-3’ (forward) and 5’- CCTCTAACGCAGCTGACCTC-3’ (reverse); -1.0kb (-1003bp~-860bp), 5’-TTGTCATTTTGGAGCCACAG-3’ (forward) and 5’-GGGCTCAGAGAAGTCTGGTG-3’ (reverse); -0.31kb (-314bp~-214bp), 5’- GGGGCTCATTCTAACAGTGC-3’ (forward) and 5’-GACACATTTCCCCACGAAGT-3’ (reverse); +0.22kb (+216bp~+268bp), 5’-CGTGTTCGCGGGTGTGT-3’ (forward) and 5’- CATTCACCTGCCGCAGAAA-3’ (reverse).

## Results

### Gene expression profiles of INO80-knockdown HeLa cells reveal potential functional versatility and complexity

**Fig 1:**
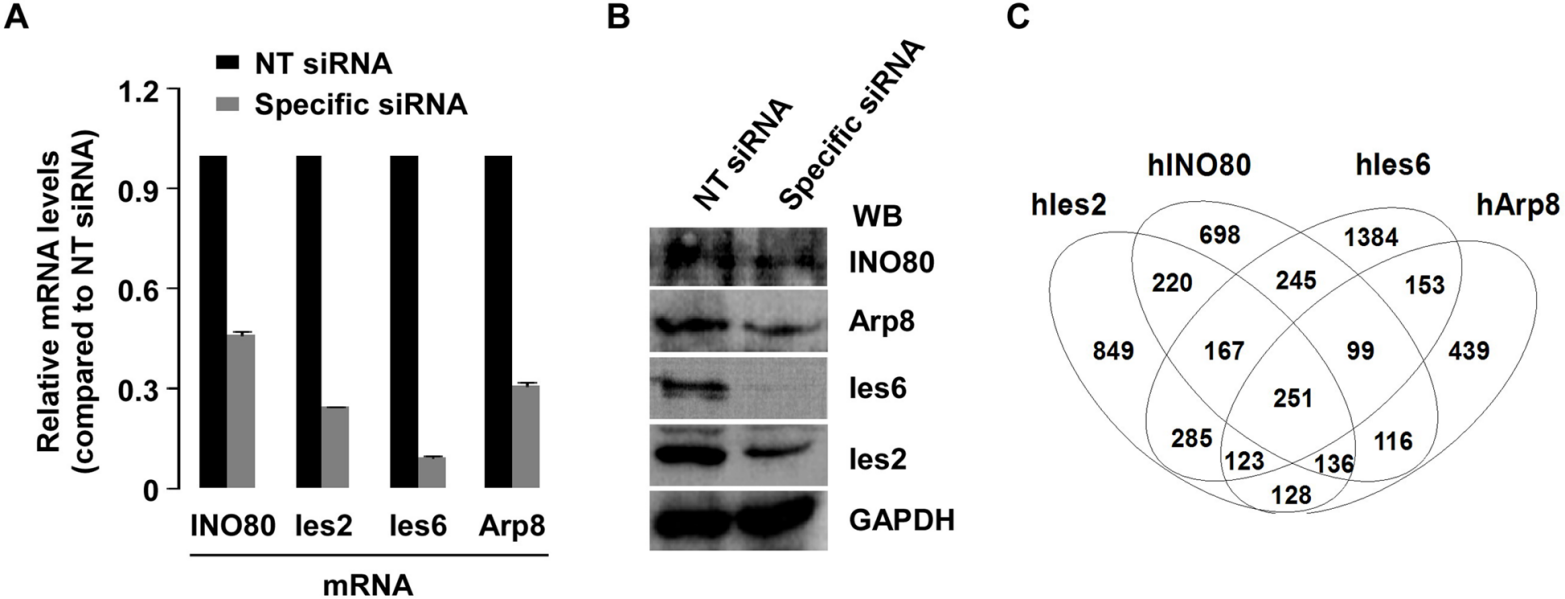
Analysis of gene expression profiles in INO80-, hIes2-, hIes6-, and hArp8- siRNA knockdown HeLa cells. To verify the siRNA knockdown efficiency, cells were transfected with 20pmol indicated siRNAs and non-targeting siRNA (siNT, as control). 48 hours after transfection, cells were harvested and lysed for immunoblotting, RT-qPCR, and DNA microarray analysis (DNA microarray was performed once). Total RNA was isolated with Trizol (Invitrogen), and the specific gene expression (all signals normalized to siNT) was measured by RT-qPCR (**A**). Whole-cell extract (WCE) was prepared by adding 4 × SDS loading buffer. Specific proteins were detected by western blot (WB) with indicated antibodies (**B**). Overlapping of differentially expressed genes (DEGs) in INO80-, hIes2-, hIes6- and hArp8-siRNA knockdown HeLa cells. DEGs between the knockdown samples and siNT control were assessed by the Illumina Custom differential expression algorithm that was conducted by software package Genomestudio V2011. Then, entrez gene IDs were exploited for Venn diagram plotting including 2159 genes for Ies2, 1936 genes for INO80, 2707 genes for Ies6, and 1445 genes for Arp8 (**C**).

We previously identified a human ATP-dependent chromatin remodeling complex which functions in gene transcription [2,3]. Although increasing evidence suggests that INO80 chromatin remodeling complex plays a key role in gene transcriptional regulation [33, 34], the exact regulatory mechanisms are still unclear. In order to identify which potential target genes were specifically regulated *via* knocking down the core components of the INO80 complexes, HeLa cells with specific siRNA (siINO80, siArp8, siIes2 and siIes6) transfections were sent to EMTD Science and Technology Development Co., Ltd. (Beijing, China) for gene expression profile analyses. The efficiency of siRNA knockdown was confirmed by RT-qPCR (**Fig 1A**) and Western blots (**Fig 1B**). To analyze the difference between differently expressed genes (DEGs) in each siRNA knockdown cells, a Venn diagram was utilized to identify overlapping genes (**Fig 1C**). Interestingly, a total of 251 genes including 149 down- and 102 up-regulated genes were co-regulated by INO80, hIes2, hIes6, and hArp8. To gain insight into whether INO80 complex was involved in important cell functional pathways by regulating pathway-related genes, a total of 2144 DEGs in at least two knocked down subunits were used for KEGG annotation by using DAVID web annotation tool. After KEGG pathway enrichment analysis, the DEGs were mainly enriched in 12 KEGG pathways including p53 signaling pathway, pathways in cancer, adherens junction, and cell cycle (**Table 2**).

**Table 2:** The enriched KEGG pathway of DEGs.

Category	Term	Description	Count	P-value	Size
KEGG	hsa04115	p53 signaling pathway	21	5.89E-07	68
KEGG	hsa05200	Pathways in cancer	47	5.57E-04	328
KEGG	hsa05222	Small cell lung cancer	16	0.005016	84
KEGG	hsa04520	Adherens junction	15	0.005605	77
KEGG	hsa04110	Cell cycle	20	0.010399	125
KEGG	hsa04510	Focal adhesion	28	0.013353	201
KEGG	hsa04010	MAPK signaling pathway	34	0.021703	267
KEGG	hsa05130	Pathogenic Escherichia coli infection	11	0.022704	57
KEGG	hsa04120	Ubiquitin mediated proteolysis	20	0.025782	137
KEGG	hsa04662	B cell receptor signaling pathway	13	0.026162	75
KEGG	hsa04512	ECM-receptor interaction	14	0.027162	84
KEGG	hsa05210	Colorectal cancer	14	0.027162	84

**Notes:** Count: the number of DEGs. Size: the total number of genes in the pathway

**Abbreviations:** DEGs, differentially expressed genes; KEGG, Kyoto Encyclopedia of Genes and Genomes.

### INO80-knockdown prolongs progression of G2/M to G1 phase in 293T cells

**Fig 2:**
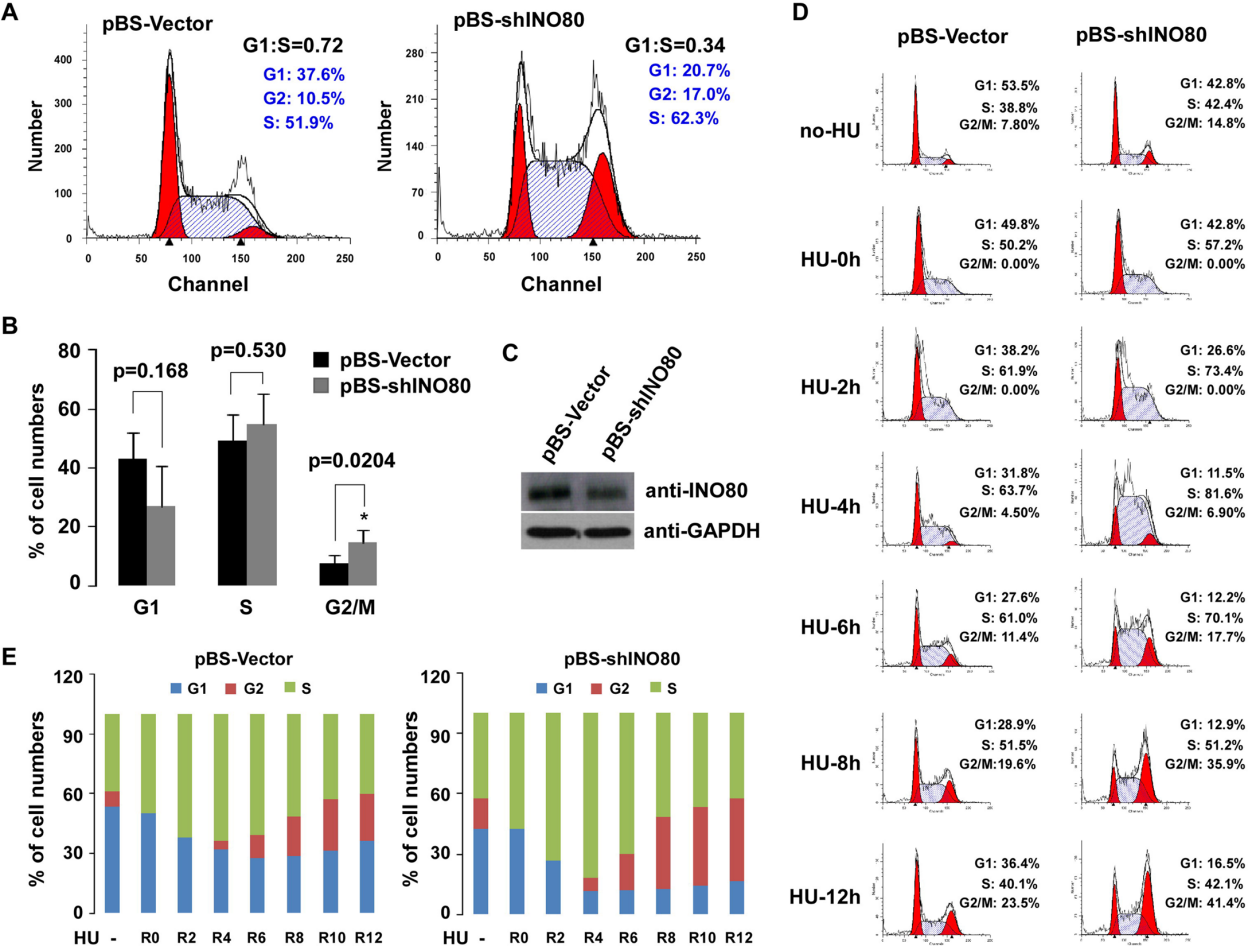
Prolonged progression of G2/M to G1 occurred in INO80-knockdown 293T cells. (**A**) Flow cytometry analysis of INO80-knockdown 293T cell cycle. 293T cells were transfected with pBS-shINO80 plasmids and pBS-Vector (as control). 48 hours after transfection, cells were harvested by trypsinization. Fluorescence-activated cell sorting (FACS) analysis of propidium iodide-stained cells was performed. (**B**) Analysis of the subpopulation of cells in cell cycle phases G1, S and G2/M. *p < 0.05 in comparison with pBS-Vector control (Student *t* test). (**C**) Verification of pBS-shINO80 knockdown efficiency. Prepared WCE from pBS-shINO80 (2 μg/well/6-well plate) treated 293T cells was subjected to SDS-PAGE (6% or 12% gel), and specific proteins were detected by WB with indicated antibodies. (**D**) Delayed progression of G2/M to G1 phase in pBS-shINO80 treated cells. pBS-shINO80 or pBS-Vector transfected 293T cells were synchronized by treatment with 1mM HU for 24 hours incubation. Cells were harvested by trypsinization 0, 2, 4, 6, 8, and 12 hrs after removal of HU. Acquired data was analyzed using ModFit LT software (Verity Software House). (**E**) Quantified cell cycle distribution in pBS-shINO80 or pBS-Vector transfected 293T cells.

Since the DEGs were linked to cell cycle according to KEGG pathway enrichment analysis, we speculated that the INO80 complex was involved in cell cycle progress. Thus, cell flow cytometry analysis was performed in INO80-knockdown cells. FACS analysis of propidium iodide-stained cells is shown in **Fig 2A**. Compared to pBS-control cells, knock down of INO80 with pBS-shRNA delayed G2/M to G1 progression. Percentage of subpopulation of cells in cell cycle phases G1, S, and G2/M are shown in **Fig 2B**. The population in cell cycle phase G2/M in INO80-knockdown cells was statistically increased compared to those in pBS-control cells (*p<0.05). To further confirm this observation, INO80-knockdown in 293T cells (**Fig 2C**) were treated with 1mM HU to block cells at G1/S phase so that no new G2/M cells could be generated. The gradual increase of the G2/M phase peak was used to evaluate the dynamics of the G2/M to G1 progression. The results of the G2/M to G1 progression after cells released from G1/S arrest by HU are shown in **Fig 2D**. Undoubtedly, silencing of INO80 produced delayed G2/M to G1 progression. As shown in **Fig 2E**, compared to pBS-control cells, the gradual increase of G2/M phase was found in shINO80-transfected cells, suggesting INO80 is involved in maintaining cell cycle progress.

### Cell cycle related gene CDKN1A (p21^Waf1/Cip1^) is regulated by INO80 chromatin remodeling complex

**Fig 3:**
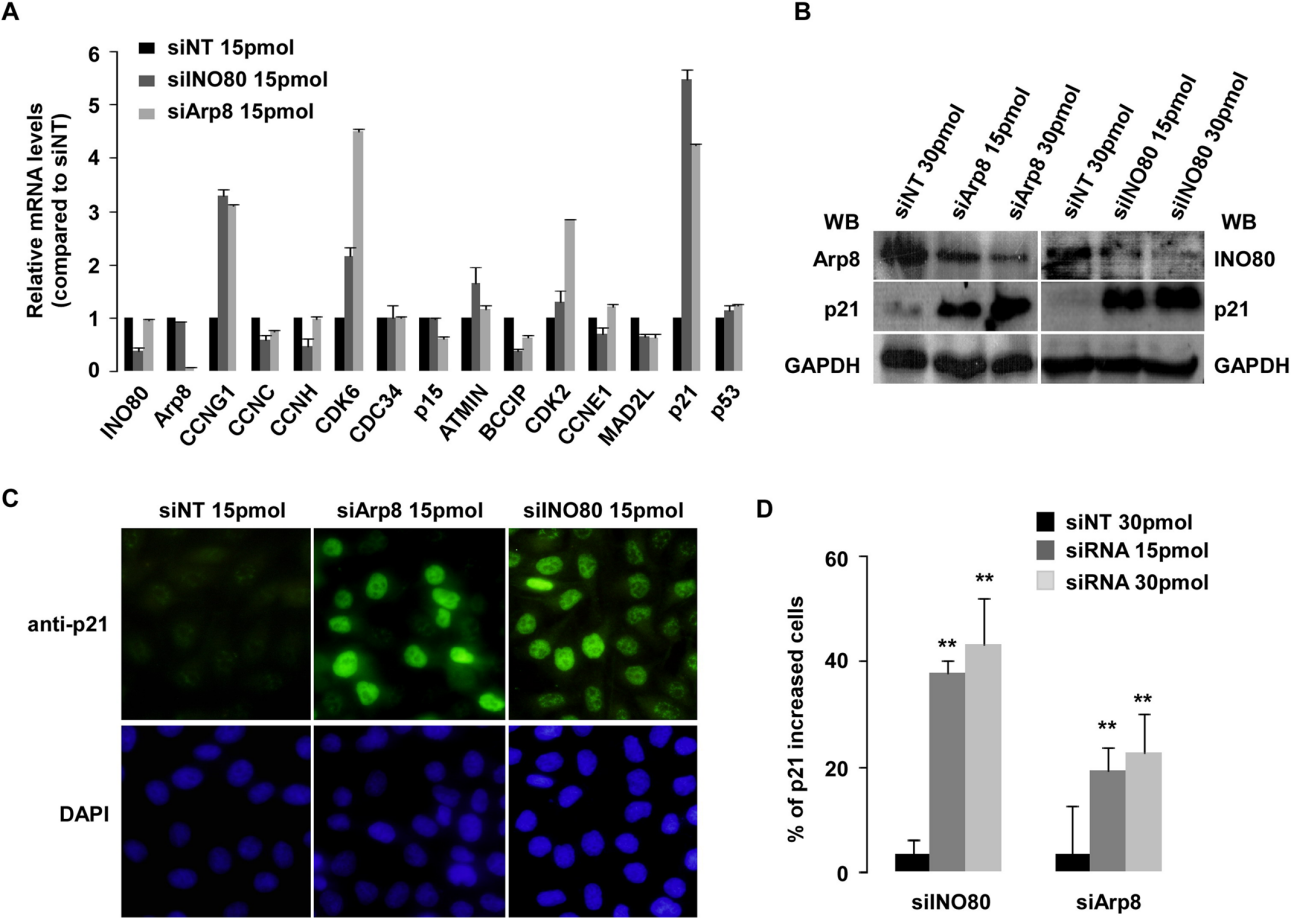
Up-regulation of CDKN1A (p21^Waf1/Cip1^) was observed in INO80 complex knockdown HeLa or 293T cells. (**A**) Verification of the mRNA of select genes from gene expression profiles. HeLa cells were transfected with 15pmol INO80- or hArp8-siRNA and siNT (as control). 48 hours after transfection, cells were harvested, and select genes from gene expression profiles were assessed by RT-qPCR. Bar graphs show ratios of RT-qPCR signals to GAPDH (all signals normalized to siNT). Error bars represent the standard error of the mean of 3 independent experiments. (**B**) Elevation of p21 protein expression in INO80- or hArp8-siRNA knockdown 293T cells. Cells were treated with indicated siRNA. 48 hours later, proteins were detected with WB using indicated antibodies. (**C**) High expression of p21 protein in cells as assessed by immunofluorescence. INO80- or hArp8-siRNA (15 or 30pmol) and siNT control was transfected into HeLa cells. 48 hours after transfection, immunofluorescence staining was performed. DAPI staining shows total nuclei. p21-positive cells were counted, and the percentage in the total cell numbers was represented in bar graph (**D**). Error bars represent the standard error of the mean of 2 independent experiments. **p < 0.01 in comparison with siNT control (Student *t* test).

To further confirm the involvement of INO80 complex in cell cycle process, select genes that were significantly up- or down-regulated in gene expression profiles were measured using RT-qPCR, with results shown in **Fig 3A**. The mRNA levels of cyclin C (*CCNC*), BRAC2/CDKN1A interacting protein (*BCCIP*) and MAD2 mitotic arrest deficient like 1 (*MAD2L1*) were decreased, while the gene expression levels of cyclinG1 (*CCNG1*), cyclin-dependent kinase 2 (*CDK2*), cyclin-dependent kinase 6 (*CDK6*) and *p21* were increased in both INO80- and hArp8-knockdown HeLa cells. **Table 3**shows a comparison of Illumina fold change and RT-qPCR fold change. Note that changes in mRNA levels of CDK inhibitors, in particular p21, emerged after microarray studies. To clarify this result, Western blot and immunofluorescence analyses were carried out in INO80- and Arp8-knockdown HeLa cells. In both protein expression assays, we observed an apparent high protein expression of p21 in INO80- or Arp8-knockdown cells (**Fig 3B and 3C**). Obvious p21 increased cells accounted for 37.8–43.3% and 19.2–22.7% of the total cell numbers in INO80- and Arp8-knockdown cells, respectively (**Fig 3D**).

**Table 3:** Comparison between the Illumina fold-change and RT-qPCR fold-change.

Gene	Gene	Gene	fold-change (siArp8)		fold-change (siIno80)	
Symble	accession No.	description	Illumina	RT-qPCR	Illumina	RT-qPCR
**CCNG1**	NM_199246	Cyclin G1	**1.35**	**3.45**	**2.36**	**3.29**
CCNC	NM_001013399	Cyclin C	0.84	-1.53	0.53	-0.80
CCNH	NM_001239	Cyclin H	1.12	-2.17	0.42	-0.06
**CDK6**	NM_001259	Cyclin-dependent kinase 6	**3.31**	**2.24**	**3.18**	**4.34**
CDC34	NM_030771	Coiled-coil domain containing 34	1.03	0.02	1.09	-0.01
CDKN2B	NM_078487	Cyclin-dependent kinase inhibitor 2B	0.62	-0.06	0.63	-1.38
ATMIN	NM_015251	ATM interactor	1.56	1.43	3.41	0.43
BCCIP	NM_016567	BRCA2 and CDKN1A interacting protein	0.83	-2.81	0.45	-1.37
**CDK2**	NM_001798	Cyclin-dependent kinase 2	**1.30**	**0.76**	**1.44**	**3.03**
CCNE1	NM_001238	Cyclin E1	1.05	-1.03	1.38	0.54
MAD2L1	NM_002358	MAD2 mitotic arrest deficient-like 1	0.81	-1.23	0.43	-1.32
**CDKN1A**	NM_078467	Cyclin-dependent kinase inhibitor 1A	**2.09**	**4.91**	**3.61**	**4.17**

**Fig 4:**
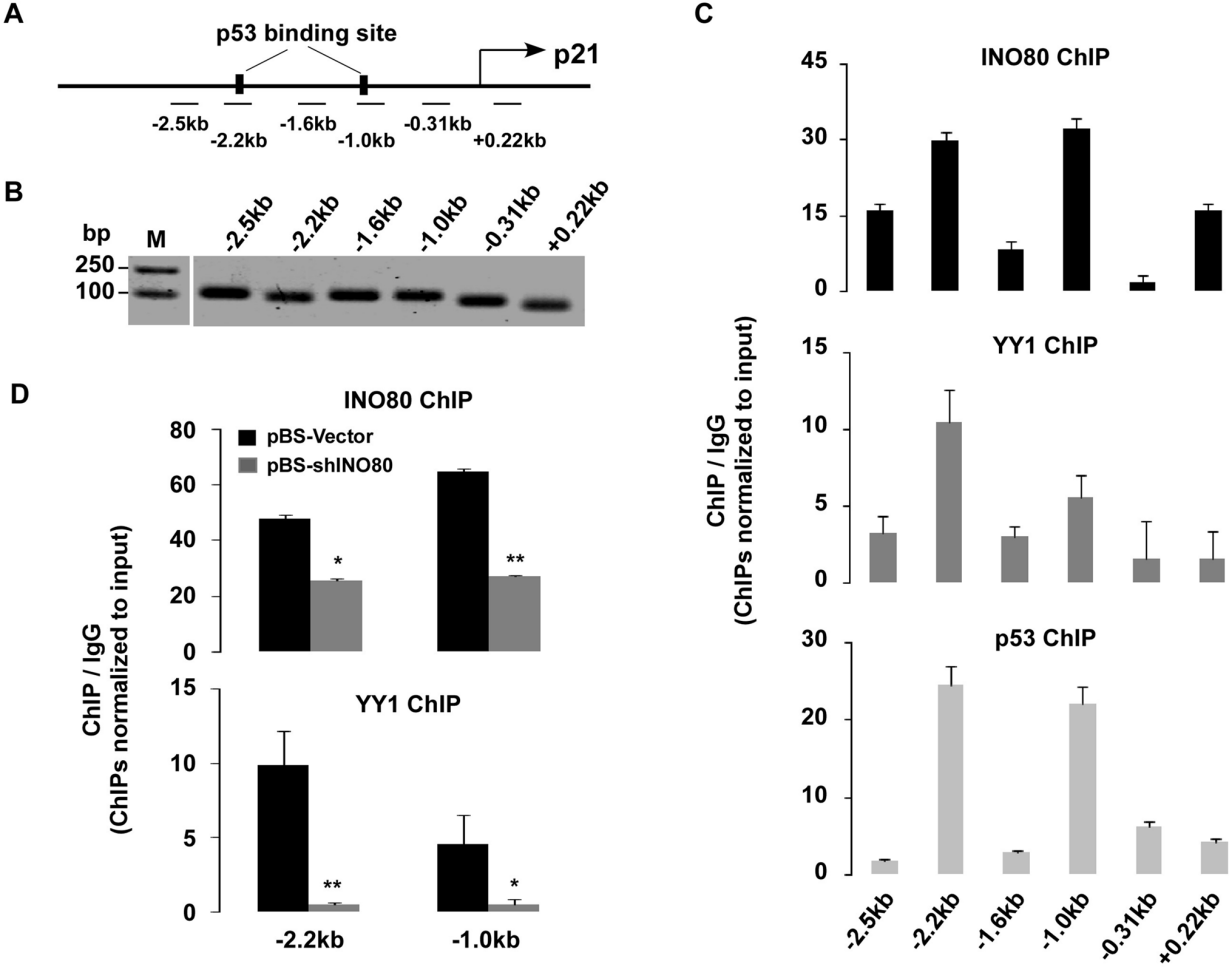
INO80 complex binds two p53 binding sites on the p21 promoter region in 293T cells. (**A**) Six primer sets in the p21 locus used for amplifying ChIP’d DNA. (**B**) **q**PCR products from each primer set were subjected to 2.5% agarose gel and visualized by ethidium bromide. (**C**) Co-occupying of the INO80 complex and p53 at the -2.2kb and -1.0kb upstream of the p21 transcriptional start site. ChIP assays were performed using INO80, YY1, and p53 antibodies in 293T cells. ChIP’d DNA was analyzed by qPCR. Bar graphs show the ratios of ChIP’d DNA signals to IgG (all signals normalized to input). Error bars represent the standard error of the mean of 3 independent experiments. (**D**) Validation of the recruitment of the INO80 complex at the *p21* promoter region. pBS-Vector and pBS-shINO80 transfected 293T cells (48 hours) were used in ChIP assays. ChIP’d DNA with INO80 and YY1 antibodies was analyzed by qPCR. Relative-fold enrichment vs IgG at the -2.2kb and -1.0kb upstream of the p21 transcriptional start site was displayed as bar graphs (all signals normalized to input). Error bars represent the standard error of the mean of 3 independent experiments.

The above experimental results clearly suggested that p21 is regulated by INO80 chromatin remodeling complex. In order to determine whether the effect of INO80 on cell cycle progress was due to its regulation of the *p21* gene transcription, the recruitment of INO80 complex to *p21* gene was studied. Six primer sets in the p21 locus were used for amplifying ChIP’d DNA (**Fig 4A**). The specificity of each primer sets was confirmed by qPCR and 2.5% DNA agarose (**Fig 4B**). ChIP assays were performed using INO80, YY1 (a subunit of INO80 complex) and p53 antibodies in 293T cells. Interestingly, INO80, and YY1 in conjunction with p53, co-occupied the -2.2kb and -1.0kb upstream of the *p21* transcriptional start site (**Fig 4C**). To further clarify whether INO80 complex indeed enriched at the -2.2kb and -1.0kb upstream of the *p21* transcriptional start site, ChIP assays using INO80 and YY1 antibodies in 293T cells with or without pBS-shINO80 transfection were carried out. As expected, silencing INO80 significantly reduced both of INO80 and YY1 binding around the p21 transcriptional start site (-2.2 kb and -1.0 kb). (**Fig 4D**).

### INO80-mediated upregulation of p21 may be in a p53-dependent manner

**Fig 5:**
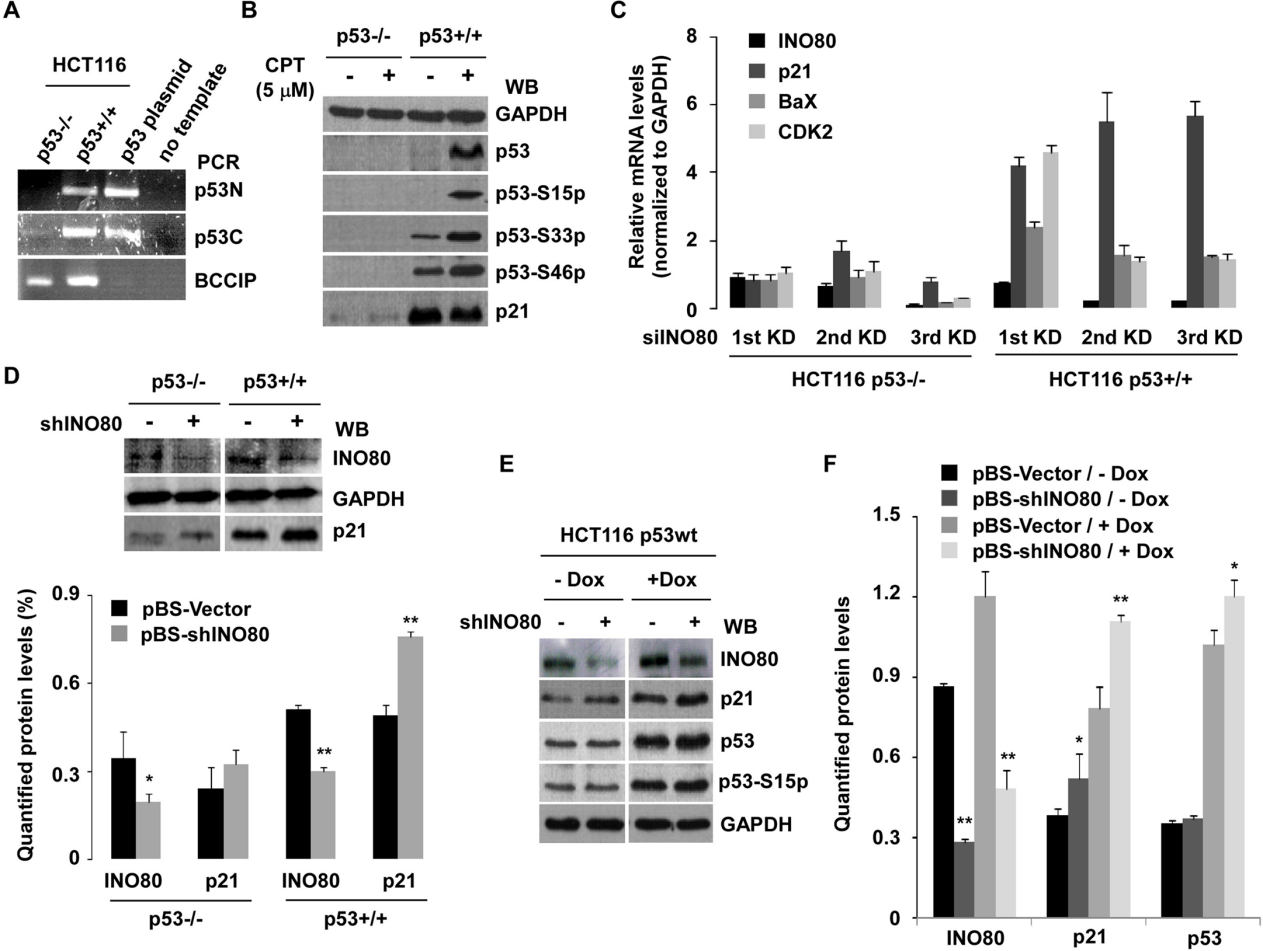
Up-regulation of p21 by INO80-knockdown is evident in p53+/+, but not in p53-/- HCT116 cells. (**A**) Clarification of p53 expression in p53+/+ or p53-/- HCT116 cell lines by PCR. Reversed cDNA as template, the p53 gene was amplified by PCR with p53 N-terminal or p53 C-terminal specific primer sets (See **Table 1**). BCCIP is the PCR control, and p53 plasmid is the positive control of the PCR product. No template in the reaction is the negative control. (**B**) Western blot analysis. Both cell lines were cultured in IMDM medium containing 5μM CPT for 8 hours. WCE was then prepared, and the proteins were detected with indicated antibodies. (**C**) High expression of *p21* gene in three consecutive INO80-knockdown cells. Both HCT116 cells were transfected with 20pmol INO80 and NT (as control) siRNAs three times every 48 hours. Then, 48 hours after each transfection, cells were harvested for RT-qPCR to assess indicated gene expression. Bar graphs show ratios of RT-qPCR signals to GAPDH (all signals normalized to siNT). (n = 3) (**D**) Obvious up-regulation of p21 in INO80-knockdown p53+/+ HCT116 cells. HCT116 cells were transfected with 2μg pBS-shINO80 (well/6-well plate) and pBS-Vector (as control). 48 hours later, indicated proteins were detected by WB with specific antibodies. Representative results from three independent experiments are shown in the upper panel. Western blot images (n = 3) were quantified with densitometry using Quantity One Basic software (Bio-Rad). *p < 0.05, **p < 0.01 in comparison with pBS-Vector control (Student *t* test). (lower panel). (**E**) p53-dependent up-regulation of p21 in INO80-knockdown cells. p53+/+ HCT116 cells were transfected with pBS-shINO80 or pBS-Vector. 24 hrs after transfection, cells were treated with 0.5μM doxorubicin (Dox) for 24 hours. WCE was then prepared and indicated proteins were measured by WB with specific antibodies. Representative results from three independent experiments are shown. (**F**) Quantified proteins. Western blot images (n = 3) were quantified with densitometry using Quantity One Basic software. **p < 0.05, *p* < 0.01 in comparison with pBS-Vector control (Student *t* test).

According to published reports, the tumor suppressor gene *p53*, a regulator of p21, can be enriched in the specific sites of the *p21* gene. Although a slightly different distribution of p53 in the *p21* gene was reported by different research groups, two regions including -2.5–2.2kb and -1.3–1.0kb upstream of the p21 transcriptional start site are considered as the main binding sites for p53 [23,35]. Distribution of INO80 complex on the *p21* gene -2.2kb and -1.0kb upstream of the transcriptional start site suggested that the regulation of INO80 on p21 transcription may be related to p53-mediated mechanism. To address this hypothesis, p53+/+ and p53-/- HCT116 colon carcinoma cell lines were used in this experiment. The cell lines were confirmed by RT-PCR (**Fig 5A**) and Western blot analysis. The induction of total p53, phosphorylated p53-S15p, p53-S33p, and p53-S46p proteins was only observed in 5μM CPT treated p53+/+ HCT116 cells (**Fig 5B**). Next, to evaluate the correlation between regulation of *p21* gene by INO80 and p53 at the transcriptional level, three consecutive (48 hours interval) INO80 knockdown with siRNA was performed in both p53+/+ and p53-/- HCT116 cells. 48 hours after each transfection, cells were harvested for RT-qPCR to assess indicated gene expression. As shown in **Fig 5C**, high expression of p53 target genes, in particular p21, remained continuously higher in three consecutive INO80 knockdown p53+/+ HCT116 cells. In contrast, there was no significant increase of gene expression including p21 detected in p53-/- HCT116 cells after INO80 knockdown.

RNAi approaches in two kinds of HCT116 cells further argued that a role for INO80 in regulation of p21 may be associated with p53-mediated mechanism. Silencing of INO80 by pBS-INO80 shRNA (2 μg/well/6-well plate) led to an obvious increase in p21 protein level in p53+/+ HCT116 cells(*p*<0.01), but not in p53-/- HCT116 cells(*p*>0.05) (**Fig 5D**). To further understand the p53-mediated functions of INO80 in regulation of p21, pBS-shINO80 or pBS-Vector transfected p53+/+ HCT116 cells were treated with 0.5μM doxorubicin (Dox) for 24 hours, and Western blot analysis were performed with the indicated antibodies (**Fig 5E**). In agreement with other reports [36], Dox induced high expression of p53 including phospho-modified p53 and p21 proteins (right panel). Note that the expression of p21 protein was higher in INO80-knockdown cells than those in pBS-control cells regardless of treatment (*p*<0.01) or non-treatment with Dox (*p*<0.05), suggesting that up-regulation of p21 by knocking down INO80 may be regulated in a p53-mediated mechanism (**Fig 5E and 5F**).

**Fig 6:**
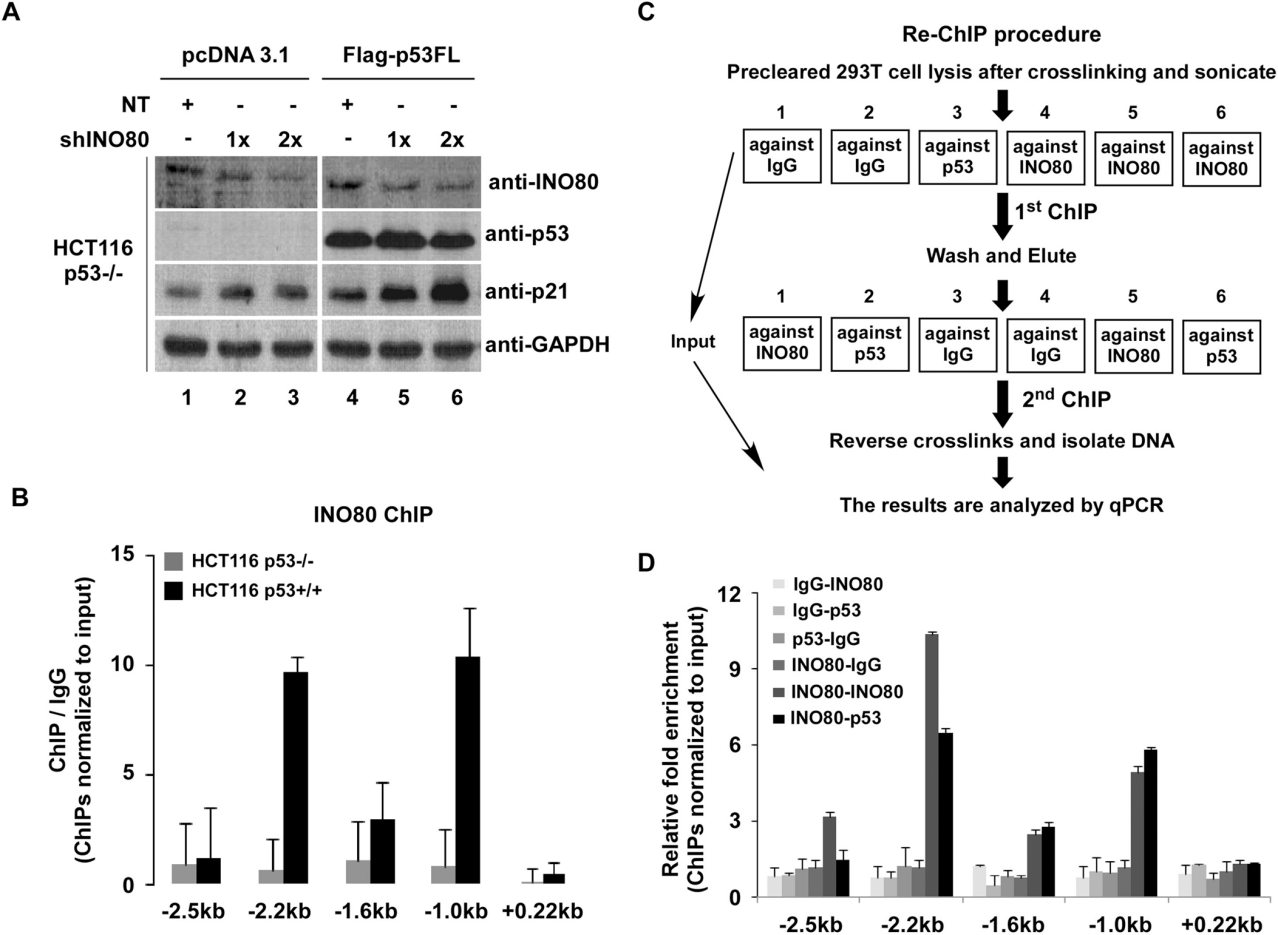
Negative regulation of p21 by INO80 complex is in a p53-dependent manner. (**A**) Significant up-regulation of p21 by shINO80 in p53FL over-expressed p53-/- HCT116 cells. p53-/- HCT116 cells were co-transfected with 0.5 μg p53FL pcDNA (pcDNA3.1 as control) and 1.0 or 2.0 μg pBS-shINO80 plasmids. 48 hours transfection, proteins were analyzed by WB with indicated antibodies. (**B**) Binding of INO80 to *p21* gene in p53+/+ HCT116 cells. ChIP assays were performed using INO80 antibody in p53+/+ or p53-/- HCT116 cells. ChIP’d DNA was analyzed by qPCR. Bar graph shows the ratios of ChIP’d DNA signals to IgG (all signals normalized to input). Error bars represent the standard error of the mean of 2 independent experiments. (**C**) Re-ChIP Experimental procedure. (**D**) Binding of INO80 and p53 to the same promoter region of *p21* gene in 293T cells. A sequential ChIP according to the experimental procedure shown in **C** was carried out using INO80 and p53 antibodies in 293T cells. ChIP’d DNA was analyzed by qPCR (all ChIPs were normalized to input). Bar graphs show the relative fold enrichment of the INO80 and p53 vs IgG. Error bars represent the standard error of the mean of 2 independent experiments.

To further confirm this hypothesis, pBS-Vector- or pBS-shINO80-treated p53-/- HCT116 cells were transiently transfected with p53 or pcDNA3.1 plasmid. As shown in **Fig 6A,** although a slight heightened expression of p21 appeared in transfection of pcDNA3.1 in INO80 knocked down p53-/- HCT116 cells (lane 1–3) (*p*>0.05 compared to NT control, data not shown), transient transfection of p53 plasmids (lane 4–6) caused evident increase of p21 in a dose-dependent manner (lane 5–6), In order to clarify whether the p53 was required for the localization of INO80 to p53 binding sites in p21, another ChIP assay was performed using INO80 antibody in p53+/+ and p53-/- HCT116 cells. As shown in **Fig 6B**, once again the distribution of INO80 was confined to-2.2 kb and -1.0 kb upstream of the p21 in p53+/+ HCT116 cells, but the peaks of distribution of INO80 on p21 disappeared in p53-/- cells, suggesting that the existence of p53 is necessary for the recruitment of the INO80 complex to the p21 promoter. To further determine the co-occupancy of the INO80 and p53 in the *p21* promoter region, a sequential ChIP (Re-ChIP) was performed based on the previous report [32]. The experiment process is shown in **Fig 6C**. As shown in **Fig 6D**, relative fold enrichment of the INO80 and p53 to the *p21* promoter (-2.2kb and -1.0kb) was only observed in INO80-INO80 and INO80-p53 Re-ChIP, suggesting that INO80 and p53 bind to the same locus of the *p21* promoter.

### Roles of INO80 in chromosome stability and cytokinesis

**Fig 7:**
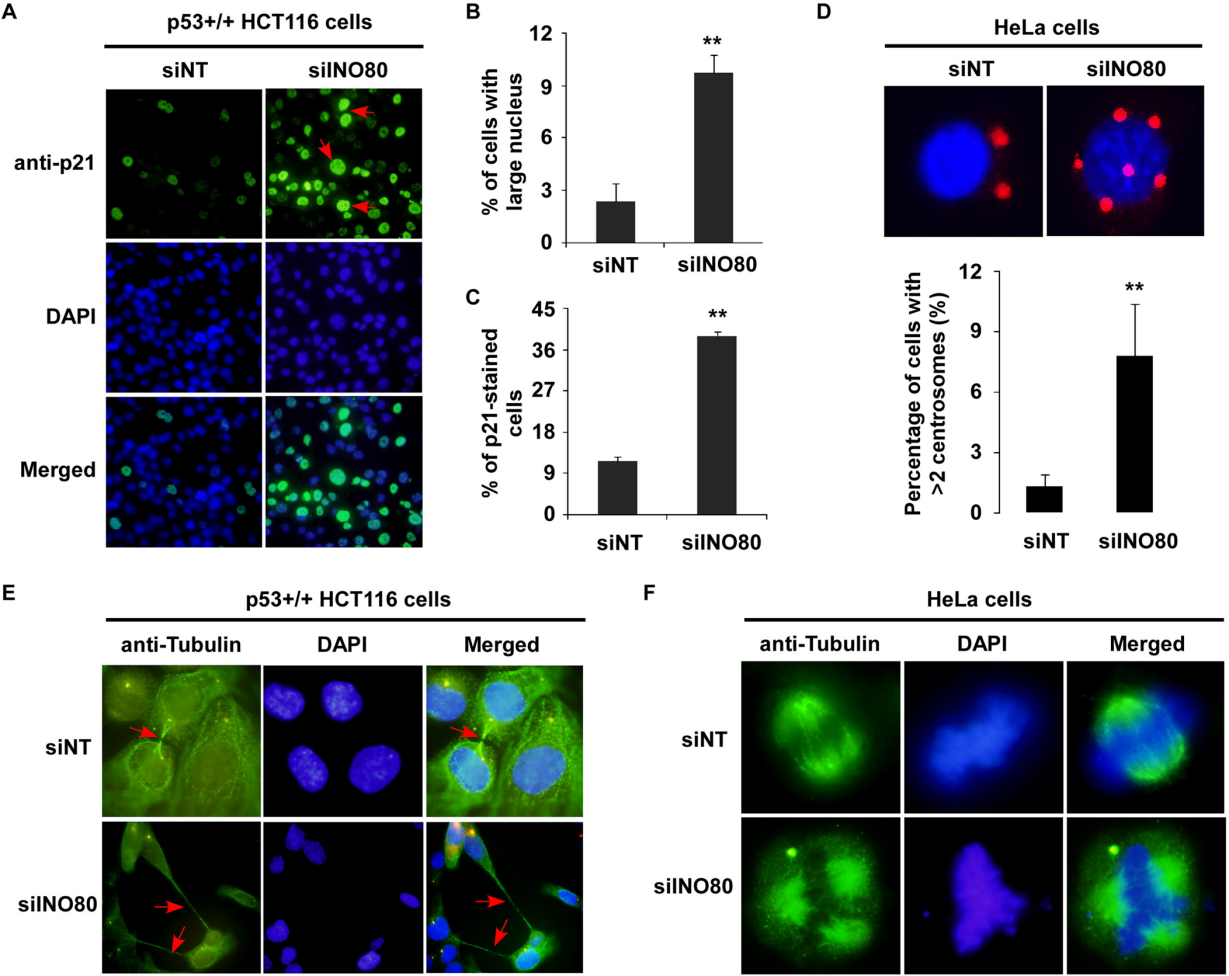
Abnormal chromosome stability resulted from INO80-knockdown in p53+/+ HCT116 or HeLa cells. (**A**) Morphological changes in siINO80 knockdown p53+/+ HCT116 cells. Representative large nuclei cells were stained with anti-p21 (green). Arrows indicate p21-stained cells with large nuclei. DAPI staining shows total nuclei. (**B**) Percentage of cells with morphology change. Obvious large nuclei cells in siNT and siINO80 cells were counted, and the percentage in the total cell numbers are represented in the bar graph. Error bars represent the standard error of the mean of three independent experiments. More than 400 cells were scored from each experiment. **p < 0.01 in comparison with siNT-control. (**C**) Number of p21-stained cells. More than 400 cells were counted from each group. **p < 0.01 in comparison with siNT-control. (**D**) Percentage of cells with more than two centrosomes per nucleus. Centrosomes in INO80 knockdown HeLa cells were stained with anti-pericentrin (red), and nuclei with DAPI (blue) (upper panel). Percentage of cells with more than two centrosomes per nucleus is shown in the lower panel. Error bars represent the standard error of the mean of two independent experiments. More than 200 cells were scored from each experiment. **p < 0.01 in comparison with siNT-control. (**E**) Cytokinesis failure in INO80-knockdown p53+/+ HCT116 cells. Cells were stained with anti-tubulin (green). (**F**) Multipolar spindle formations in INO80 knockdown HeLa cells. Mitotic spindle was stained with anti-α-tubulin.

It has been reported that p21, a potent cyclin-dependent kinase inhibitor, plays an important role in regulating cell cycle phase G2/M [37,38]. Silencing of INO80 resulted in both up-regulation of p21 and cell cycle G2/M phase arrest, suggesting that INO80 complex might block the progression of G2/M to G1 by regulating p21. To gain a clear idea of the roles of INO80 complex in G2/M phase, the impact of INO80 on chromosome stability in p53+/+ HCT116 and HeLa cells were studied. Knockdown of INO80 with siRNA in p53+/+ HCT116 cells led to an increase of large nuclei cells (**Fig 7A**). Significant elevation of large nuclei cells was found in INO80-knockdown cells (**Fig 7B**, *p* < 0.01). Again, increased p21-stained cells were observed in INO80-silenced cells (**Fig 7C**, *p* < 0.01). Interestingly, high-expressed p21 was observed in most of the large nuclei cells, indicating the roles of INO80 in chromosome stability might be mediated by regulating p21. In addition, significant increases of cells with more than two centrosomes per nucleus were observed in INO80-knockdown cells (**Fig 7D**). Moreover, INO80-knockdown also resulted in cytokinesis failure (**Fig 7E**) and multipolar spindle formations (**Fig 7F**).

## Discussion

In this report we first present evidence that the INO80 chromatin remodeling complex negatively regulates the p21 expression in a p53-mediated mechanism using *in vitro* biological experiments combined knockdown/over-expression approaches. In addition, ChIP and Re-ChIP assays indicated that the recruitment of INO80 complex in the *p21* gene relied on the existence of p53. By combining methods of flow cytometry and immunofluorescence staining, we postulate that the role of the INO80 complex to maintain the normal cell cycle and chromosome stability is at least partly related to the regulation of p21.

Ino80 complex, a member of the SWI/SNF2 superfamily of chromatin remodeling complexes, is highly conserved from yeast to human [39]. Except for a set of highly evolutionarily orthodox core subunits, six metazoan-specific subunits and YY1 are identified in the INO80 complex [2,3]. Interestingly, all of the metazoan-specific subunits are composed of an N-terminal regulatory module. The other subunits form two modules assembled on the conserved HSA/PTH and ATPase domains of the INO80 protein, therefore catalyzing ATP-dependent nucleosome remodeling activity of the INO80 complex [12,13]. Although the exact functions of Ino80 HSA/PTH domains remain unclear, increasing evidence argues that HSA/PTH domains play an important role in cellular processes in various species. Several nuclear Arps such as Arp4 and Arp8 have been shown to bind to DNA and histones, suggesting a possibility that Ino80 HSA/PTH-containing modules may contribute to recognition of DNA and/or nucleosome substrates [14,40], thereby regulating gene expression through chromatin structure alteration. Our experimental results also support this view. Based on the analysis of performed gene expression profiles from INO80-, Arp8-, hIes2- or hIes6-knockdown HeLa cells, we clarified that the INO80 complex was involved in multiple signal pathways including cell cycle (**Table 2**), suggesting the importance of the INO80 complex in cellular biological processes.

It has been reported that the Ino80 chromatin remodeling complex contributes to a wide variety of chromatin-dependent nuclear transactions, including gene transcription; DNA replication and DNA repair [33]. Data from DNA microarray and ChIP experiments previously revealed that as many as two-thirds of the yeast genes in which transcription were affected by the Ino80 complex [41,42]. In contrast, studies of the INO80 complex on gene transcription, especially on genes related to cell cycle in human cells, are rare. Based on gene expression profiles and *in vitro* biological experiments, we observed the up-regulation of p21 by knocking down either INO80 or Arp8 in human cells (**Fig 3**). ChIP assay with available INO80 and YY1 antibodies revealed that the two binding sites of the INO80 complex in p21 gene were similar to those of p53 in p21 [23,35]. Furthermore, ChIP and Re-ChIP assays confirmed that the INO80 complex and p53 co-occupy the *p21* gene promoter (-2.2 and -1.0kb upstream of the p21 transcriptional start site), suggesting the collaborative mechanism of INO80 complex and p53 (**Fig 4C** and **Fig 6D**). As expected, further investigation verified that the up-regulation of p21 by INO80 was in a p53-dependent manner (**Figs 5 and 6**): 1) Significant increase of p21 expression was observed in INO80 knockdown p53+/+ HCT116 cells; 2) Recruitment of INO80 in *p21* gene only occurred in p53+/+ HCT116 cells, and not in p53-/- cells; 3) Obvious dose-dependent increases of p21 were produced by over-expression full length of p53 in INO80 knockdown p53-/- HCT116 cells. On the other hand, research evidence demonstrated that high-expression of p21 in response to various stimuli and stress signal can be regulated in a p53-dependent and/or p53-independent manner. For example, p21 expression is up-regulated by forkhead box A1/2 in p53-null H1299 lung carcinoma or by SP1 in p53-null Caco-2 colon carcinoma cells [43,44]. In our study, note that p21 protein also had a slight increase in INO80 knockdown p53-/- HCT116 cells (**Fig 5D** and **Fig 6A**), suggesting that p53-mediated regulation of p21 may not be the only mechanism of the INO80 complex.

According to a report by Hur SK *et al*. [4], INO80 is critical for normal cell cycle progression, particularly in S-phase progression and mitosis. In this study, although the cell cycle arrest was exhibited in both S and G2/M phases, the significant prolonged progression of cell cycle phase G2/M to G1 was found in INO80-knockdown 293T cells (**Fig 2**), indicating that the INO80 complex might be implicated in the progression from DNA replication to cell division. Consistent with this idea, we carried out cell staining with primary antibodies in INO80-knockdown p53+/+ HCT116 and HeLa cells to know the effects of INO80 on chromosome stability. Similar to that previous report [4], siINO80 treatment not only increased the large nuclei cells, but also caused multipolar spindle formations, cytokinesis failure and centrosome amplification in p53+/+ HCT116 or HeLa cells (**Fig 7**). Interestingly, hyper-expression of p21 was also observed in most of morphologically changed cells, suggesting p21-mediated function of INO80 in genome stability. It is worth noting that p21 shows bidirectional functions as both a tumor suppressor and an oncogene. In the nucleus, p21 as a negative cell cycle regulator, functions as a tumor suppressor. While in the cytoplasm, p21 acts as an oncogene by inhibiting apoptosis and facilitating cell proliferation [21,45]. In addition, phosphorylation of p53 at p53-S15p and p53-S20p can activate downstream target genes such as p21 which play an important role in G2/M checkpoint through inhibition of Cdk1/Cyclin B [46]. In this study, the presence of Dox in INO80 silencing p53+/+ HCT 116 cells also increased the protein level of p53 including p53-S15p and p21 (**Fig 5E and 5F**), suggesting the up-regulation of p21 by INO80 chromatin remodeling complex might be associated with cell cycle phase G2/M checkpoint and abnormal chromosome stability.

Although our ChIP and Re-ChIP experimental data suggest that INO80 complex inhibits the gene expression of p21 by acting as a transcriptional co-repressor of p53 in cells, the question of how to switch the transcriptional co-activator of p53 remains yet unclear. There are several possibilities we can think of at the moment: 1) Knockdown INO80 destroyed the joint action between the INO80 and p53; 2) p53 may switch to co-activator after knockdown INO80 and activate the p21 gene transcription; 3) non-p53 mediated mechanism may be involved in the transcription of p21 gene. Therefore, more experimental data is required to address those issues.

## Conclusions

In summary, given that INO80 complex performs an important function in maintaining the normal cell cycle and genome stability, it is necessary to investigate the functions of INO80 complex in tumorigenesis and cancer therapy in the future.
